# The Impact of Diet‐Induced Weight Loss on Biomarkers for Colorectal Cancer: An Exploratory Study (INTERCEPT)

**DOI:** 10.1002/oby.21984

**Published:** 2017-10-31

**Authors:** Rebecca J. Beeken, Helen Croker, Malgorzata Heinrich, Austin Obichere, Nicholas Finer, Neil Murphy, Robert Goldin, Naomi J. Guppy, Rose Wilson, Abigail Fisher, Andrew Steptoe, Marc J. Gunter, Jane Wardle

**Affiliations:** ^1^ Department of Behavioural Science & Health University College London London UK; ^2^ Leeds Institute of Health Sciences University of Leeds Leeds UK; ^3^ Colorectal Service, University College London Hospital London UK; ^4^ Centre for Obesity Research University College London London UK; ^5^ Section of Nutrition and Metabolism, International Agency for Research on Cancer Lyon France; ^6^ Department of Medicine Imperial College London London UK; ^7^ University College London Advanced Diagnostics London UK; ^8^ Cancer Research UK London UK

## Abstract

**Objective:**

The aim of this study was to explore the potential effects of diet‐induced weight loss on molecular biomarkers of colorectal cancer risk in serum and colorectal tissue.

**Methods:**

This single‐arm exploratory study included 20 adults with BMI ≥ 30 kg/m^2^ completing an 8‐week, complete, low‐energy liquid diet. Pre‐ and postintervention anthropometric measurements, fasting blood draws, and endoscopic examinations to procure colorectal biopsies were performed. Fasting insulin, glucose, insulinlike growth factor 1 (IGF‐1), C‐reactive protein (CRP), and blood lipids were measured in serum, and tissue markers of apoptosis (M30), colonocyte proliferation (Ki‐67), and insulin signaling (phospho‐mTOR) were assessed using immunohistochemical staining.

**Results:**

Participants achieved substantial weight loss (mean = 13.56%). Mean concentrations of insulin, glucose, and cholesterol were significantly reduced (*P* < 0.05), but IGF‐1 and CRP were not. Colorectal tissue expression of Ki‐67 was significantly reduced (preintervention mean score = 7, postintervention mean score = 3.9, mean % change −43.8; *P *= 0.027). There were no significant changes in M30 or phospho‐mTOR.

**Conclusions:**

Weight loss in individuals with obesity was associated with improvements in insulin sensitivity and blood lipid profiles and a significant reduction in tissue Ki‐67 expression. This is one of the first studies to demonstrate potential cancer‐relevant changes in colorectal tissue following weight loss achieved through diet.

## Introduction

Colorectal cancer (CRC) is the fourth most commonly diagnosed malignancy in the United Kingdom, with 41,266 new cases diagnosed in 2014 and 15,903 deaths 1. Risk of CRC is increased significantly in individuals with obesity (BMI ≥ 30 kg/m^2^) 2. With more than a quarter of adults in the United Kingdom now classified as having obesity 3, this is a potentially growing source of CRC cases 4. Consequently, it is important to establish whether weight loss can mitigate development of this malignancy.

Bariatric surgery, which leads to significant weight loss in individuals with obesity, has been associated with a reduction in CRC 5, 6, though not all studies have reported reductions in risk 7, 8, 9. However, there are no comparable data for dietary weight loss programs 10. Weight loss achieved by nonsurgical methods is often poorly maintained in the long term, so investigation of the effects of interventions based on diet‐induced weight loss on cancer outcomes is difficult and requires very large cohorts with long follow‐up periods 11. An alternative approach is to investigate the effects of weight loss on molecular pathways and biomarkers that have been implicated in CRC development. Weight loss achieved through low‐energy (∼800 calories per day) meal replacement diets offers an ideal paradigm for such investigations because the weight loss achieved is typically rapid and substantial when adherence is good 12.

There is increasing consensus that the insulin/insulinlike growth factor 1 (IGF‐1) axis and related inflammatory pathways play a significant role in the obesity‐CRC link 13, 14, 15, 16, 17. Animal models of CRC have shown significant modifications in these pathways following calorie restriction 18. In humans, intentional weight loss and bariatric surgery yield favorable changes in circulating concentrations of insulin and inflammatory factors, such as C‐reactive protein (CRP), though changes in IGF‐1 are less consistent 19, 20. However, few studies have directly assessed the effect of weight reduction on colorectal tissue.

A study in which 10 women with obesity achieved 10% weight loss following a meal replacement diet found reductions in circulating and rectal tissue concentrations of two markers of inflammation: tumour necrosis factor alpha (TNF‐α) and interleukin 8 (IL‐8) 21. Several randomized controlled trials of physical activity have also demonstrated significant effects of exercise on both serum markers and colon expression of proliferative markers and apoptotic factors 22, 23, 24. Furthermore, a recent study found changes in Ki‐67 (an established marker of cell proliferation that has been identified as potentially useful biomarker of cancer risk 25, 26) in colon tissue following a change in diet, but the study did not measure weight changes 25. These findings suggest that interventions that modify energy balance can yield significant molecular changes at the colorectal tissue level and add support to a potential antitumorigenic effect of weight loss in the colorectum. The current analysis is a small, single‐arm study that aims to build on this research by exploring the potential effects of diet‐induced weight loss on serum markers for CRC and changes in colorectal tissue expression of markers of cell proliferation, apoptosis, and insulin signaling. The findings from this study will inform the design of future larger randomized controlled trials on weight loss and changes in CRC biomarkers.

## Methods

This was a single‐arm exploratory study to explore the potential effects of diet‐induced weight loss on molecular biomarkers of CRC risk in serum and colorectal tissue.

### Participants

Twenty individuals with obesity (BMI ≥ 0 kg/m^2^) were recruited via advertisements placed around University College London. Participants were recruited between July 2013 and July 2014. Participants were required to be fluent in English, nonsmokers, and aged between 18 and 60 years. Exclusion criteria included a history of major depression, history of cardiovascular disease, pregnancy, taking medication prescribed by a general practitioner over the past month (oral contraceptives were allowed), diabetes, or other diseases (liver, kidney, heart, lung, blood, or skin). Potential participants were also excluded if they had previously had intestinal surgery, a history of malabsorption, or regularly used drugs with anti‐inflammatory or hypoglycaemic properties, as these would interfere with the assessment of insulin‐related parameters. Finally, individuals with a previous diagnosis of cancer were excluded, as this could affect measured biomarkers.

### Weight loss intervention

After an initial consultation and assessment with an obesity‐specialist physician, participants followed an 8‐week liquid weight‐loss diet program (810 calories per day) based on formula diet products (Cambridge Weight Plan, Northants, UK). Previous studies have shown this to be a safe and effective dietary method for weight loss 27, 28, 29. The program was delivered by trained researchers under the supervision of a registered dietician and obesity‐specialist clinician. Support and advice on behavior change techniques were offered on a weekly basis in the form of face‐to‐face contact. Each session lasted between 30 minutes and an hour. Participants were requested to maintain their preintervention physical activity levels for the duration of the 8‐week intervention. At the end of the 8 weeks, 4 weeks of additional support were provided to help participants with meal reintroduction and weight loss maintenance.

### Study procedures

At baseline and at the 8‐week follow‐up point (i.e., on completion of the weight‐loss diet), a research nurse measured height, weight, and waist circumference for each participant. Two 20‐ml fasting blood samples were taken for assessment of serum concentrations of insulin, glucose, CRP, IGF‐1, total cholesterol, high‐density lipoprotein (HDL), and triglycerides.

Participants also underwent a flexible sigmoidoscopy at baseline and follow‐up to obtain colon and rectal biopsies. These procedures were performed by an experienced Consultant Colorectal Surgeon at the Colorectal Centre, University College London Hospital. For each participant, and at each time point, biopsies 10, 11, 12, 13, 14, 15 ∼1‐mm thick were obtained from different sections of the bowel that were accessible during flexible sigmoidoscopy (sigmoid colon, rectum) and were stored as formalin‐fixed paraffin‐embedded slides and as fresh samples stored in RNALater.

### Ethics

The study was approved by London–Harrow Research Ethics Committee (reference: 13/LO/0080) and was conducted in accordance with the Helsinki Declaration of 1975 as revised in 1983. Written informed consent was obtained from all participants before joining the study. The study was registered on the ISRCTN registry as ISRCTN35702367.

### Laboratory methods

All serologic measurements were performed at Imperial College NHS Trust Clinical Laboratories. Serum insulin, CRP, and IGF‐1 were analyzed using enzyme‐linked immunosorbent assays. Markers of apoptosis (M30), colonocyte proliferation (Ki‐67), and insulin signaling pathway activation (phospho‐mTOR) were assessed using immunohistochemical staining in the laboratory of Professor Robert Goldin, Imperial College, and Dr Naomi Guppy, University College London. All staining was performed using the Leica Bond III automated immunostaining platform on the Leica Bond Polymer Refine (Leica, DS9800) DAB polymer detection system. Ki‐67 (mouse monoclonal MIB‐1, Dako, cat. no. M7240) was applied at a dilution of 1/120 for 15 minutes at room temperature, following on‐board heat‐induced epitope retrieval (HIER) using Leica Epitope Retrieval 2 (ER2, pH9 retrieval solution; Leica, AR9640) for 20 minutes. M30 (mouse monoclonal M30 cytoDEATH, Roche, cat. no. 12 140 322 001) was applied at a dilution of 1/50 for 30 minutes at room temperature, following on‐board HIER using Leica Epitope Retrieval 1 (ER1, pH6 retrieval solution; Leica, AR9961) for 30 minutes. Phospho‐mTOR (rabbit monoclonal 49F9, Cell Signaling Technologies cat. no. 2976) was applied at a dilution of 1/50 for 20 minutes at room temperature, following on‐board HIER using Leica ER2 for 30 minutes.

### Statistical analysis

This was an exploratory study, and so no formal sample size calculation was performed. Based on the previous study, which found significant changes at the tissue level in 10 participants 21, we aimed for a sample size of 10 to 20 to enable us to demonstrate feasibility and potential effects of the weight loss intervention. The intervention effects were evaluated by exploring the differences in the geometric mean changes from baseline to 8 weeks by paired *t* tests and by generalized estimating equation modification to linear regression models to account for the longitudinal nature of the data. Serologic and tissue expression data, if not normally distributed, was log transformed to achieve normality prior to performing parametric analyses.

## Results

Participants were mostly female (70%), aged 21 to 57 years, and of white ethnicity (90%; Table 1). Participants had a mean baseline BMI of 34.0 (32.2‐36.0) and waist circumference of 102.5 (98.5‐106.7) (Table 2).

**Table 1:** Baseline characteristics

	*n*	%
**Age, y, mean (range)**	35	(21–57)
**Gender**		
**Male**	6	(30%)
**Female**	14	(70%)
**Ethnic origin**		
**White‐British**	10	(50%)
**White‐Other**	8	(40%)
**Asian/Mixed**	1	(5%)
**Other**	1	(5%)
**Highest level of education**		
**Vocational qualification/A‐level**	6	(30%)
**Degree or higher**	13	(65%)
**Other**	1	(5%)

Participants achieved clinically important and beneficial anthropometric outcomes (Table 2); weight loss was substantial, with a baseline mean of 98.8 kg reduced to a follow‐up mean of 85.4 kg (% mean change, −13.6; *P* = 0.002). Mean BMI was also reduced from 34.0 kg/m^2^ to 29.4 kg/m^2^ (% mean change, −13.6; *P* = 0.0008). Mean waist circumference was reduced by 11.9% (*P* = 0.0003).

**Table 2:** Baseline and follow‐up mean levels of serologic (*n* = 17) and tissue (*n* = 20) markers

	Baseline, mean (95% CI)	Follow‐up, mean (95% CI)	Mean % change, mean (95% CI)	*P* value
**Weight (kg)**	98.78 (92.59 to 105.39)	85.39 (79.82 to 91.34)	−13.56 (−13.73 to 13.39)	0.002
**Waist circumference (cm)**	102.54 (98.54 to 106.71)	90.32 (85.62 to 95.27)	−11.93 (−12.09 to −11.76)	0.0003
**BMI (kg/m^2^)**	34.01 (32.15 to 36.0)	29.40 (27.63 to 31.28)	−13.56 (−13.73 to −13.39)	0.0008
**Waist to hip ratio**	0.86 (0.82 to 0.90)	0.82 (0.78 to 0.85)	−5.10 (−5.26 to −4.93)	0.08
**Insulin (mIU/L)**	11.55 (8.44 to 15.82)	6.42 (5.15 to 8.00)	−44.47 (−44.66 to −44.29)	0.003
**Glucose (mmol/L)**	5.24 (4.96 to 5.54)	4.91 (4.63 to 5.21)	−6.31 (−6.48 to −6.14)	0.01
**HOMA-IR**	1.51 (1.11 to 2.06)	0.83 (0.67 to 1.04)	−44.85 (−45.03 to −44.66)	0.002
**IGF‐1 (nmol/L)**	22.27 (19.93 to 24.88)	20.69 (17.86 to 23.98)	−7.08 (−7.26 to −6.91)	0.40
**CRP (mg/L)**	2.43 (1.54 to 3.85)	1.94 (1.25 to 2.99)	−20.52 (−20.72 to −20.32)	0.44
**Total cholesterol (mmol/L)**	4.88 (4.47 to 5.32)	3.70 (3.35 to 4.10)	−24.01 (−24.18 to −23.84)	0.0001
**HDL (mmol/L)**	1.41 (1.27 to 1.56)	1.14 (1.02 to 1.27)	−19.0 (−19.18 to −18.84)	0.006
**Triglycerides (mmol/L)**	0.93 (0.78 to 1.10)	0.81 (0.72 to 0.90)	−13.0 (−13.15 to −12.81)	0.17
**Ki‐67**	7.0 (6.0 to 8.0)	3.9 (2.3 to 7.0)	−43.8 (−44.16 to −43.51)	0.027
**M30**	8.4 (2.9 to 24.2)	52.2 (5.9 to 459.5)	521.0 (−520.14 to −522.18)	0.09
**phospho-mTOR**	208.0 (148.6 to 291.0)	219.1 (172.5 to 278.5)	5.38 (−5.09 to −5.66)	0.77

We were unable to obtain blood samples pre‐ and post‐weight loss from three of the participants. For those participants for whom pre and post blood samples were available (17/20; 85%), diet‐induced weight loss resulted in statistically significant mean reductions in serum insulin (% mean change −44.5; *P* = 0.003), glucose (% mean change −6.3; *P* = 0.01), HOMA‐IR (% mean change −44.9; *P* = 0.002), total cholesterol (% mean change −24.0; *P* = 0.0001), and HDL (% mean change −19.0; *P* = 0.006). Mean concentrations of IGF‐1, CRP, and triglycerides were also reduced during the intervention, but these changes did not meet statistical significance (Table 2).

In linear mixed‐model analyses, in addition to inducing significant reductions in weight and other anthropometric parameters, the weight loss intervention induced statistically significant reductions in fasting insulin (β = −6.95; *P* = 0.002), glucose (β = −0.33; *P* = 0.01), HOMA‐IR (β = −1.81; *P* = 0.002), total cholesterol (β = −1.17; *P* < 0.001), HDL (β = −0.27; *P* < 0.001), and triglycerides (β = −0.15; *P* < 0.001) (Table 3).

**Table 3:** Linear mixed modeling analysis of the effect of the intervention on anthropometric parameters and serum biomarker levels

	Β‐coefficient (95% CI)	*P*
**Weight**	−13.34 (−14.72 to −12.0)	<0.001
**BMI**	−4.57 (−4.96 to −4.17)	<0.001
**Waist circumference**	−12.0 (−13.7 to −10.31)	<0.001
**Hip circumference**	−8.53 (−10.41 to −6.65)	<0.001
**Waist to hip ratio**	−0.04 (−0.06 to −0.02)	<0.001
**Glucose**	−0.33 (−0.56 to −0.10)	0.01
**Insulin**	−6.95 (−9.2 to −3.04)	0.002
**HOMA‐IR**	−1.81 (−3.21 to −0.75)	0.002
**IGF‐1**	−1.22 (−4.0 to −1.66)	0.38
**CRP**	−0.92 (−2.01 to −0.17)	0.09
**Total cholesterol**	−1.17 (−1.41 to −0.94)	<0.001
**HDL**	−0.27 (−0.39 to −0.02)	<0.001
**Triglycerides**	−0.15 (−0.29 to −0.65)	<0.001

**Figure 1:**
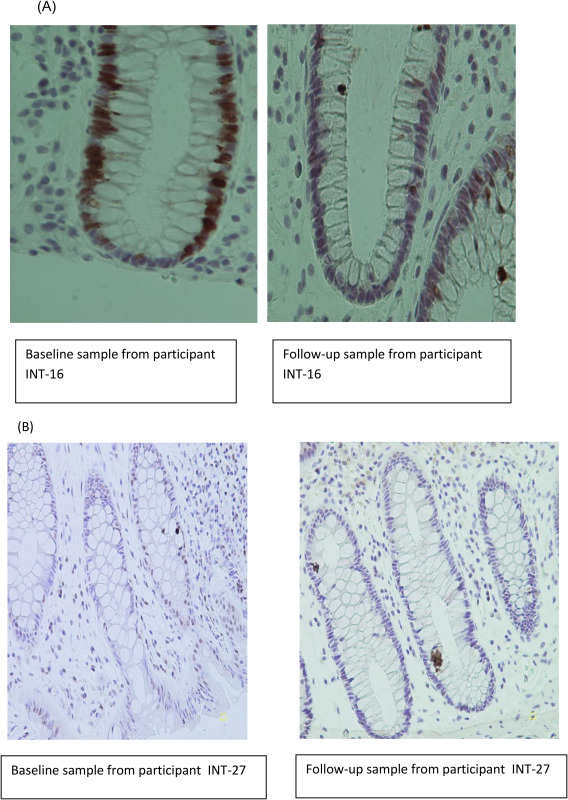
(**A**) Ki‐67 immunohistochemical staining in colorectal tissue. (**B**) M30 staining.

Tissue analyses indicated an increase in apoptosis, as assessed by M30, during the intervention (preintervention mean H‐score = 8.4, postintervention mean H‐score = 52.2, mean % change, 521.0); but the change did not attain statistical significance (*P* = 0.09). On the other hand, concentrations of Ki‐67, a proliferation marker, were significantly reduced over the intervention period (preintervention mean = 7.0; postintervention mean, 3.9; mean % change, −43.8%; *P* = 0.027; Figure 1). We did not observe any changes in tissue concentrations of phospho‐mTOR during the intervention.

## Discussion

Previous studies have demonstrated changes in serum biomarkers for CRC risk following weight loss 19, 20, but few have explored changes at the tissue level. In this exploratory study, weight loss was associated with significant improvements in insulin sensitivity and blood lipid profiles as well as a significant reduction in Ki‐67 expression in colorectal tissue. The results of this analysis provide preliminary evidence for weight‐related molecular changes in the colorectum following intentional weight loss achieved through a low‐energy meal replacement diet.

Combined with the previous study, in which a 10% diet‐induced weight loss led to reductions in circulating and rectal tissue concentrations of inflammatory markers in 10 women 21, these findings lend support for a potential antitumorigenic effect of weight loss among individuals with obesity. Ki‐67 is an established marker of cell proliferation and has been identified as a potentially useful biomarker of cancer risk 25, 26. The evidence for the use of proliferation biomarkers in humans is based on the known increase in proportion of epithelial cells engaged in DNA synthesis in premalignant conditions of the colorectum, as well as the observed expansion of the proliferative zone, causing a shift of the proliferating cells from the base to the surface of the colonic crypt. It is currently unknown at what point reductions in Ki‐67 can be considered clinically meaningful. However, the reduction in Ki‐67 expression in colorectal tissue observed in our study, alongside reductions in circulating insulin, adds support for the hypothesis that changes in the insulin signaling pathway may be an important mechanism through which diet‐induced weight loss influences cell proliferation in the colorectum, and ultimately CRC risk. Studies with larger samples are needed to confirm these findings and to explore whether changes in circulating insulin mediate the association between weight loss and reduced Ki‐67.

In our study, we did not observe a significant change in circulating concentrations of IGF‐1 following weight loss. Despite experimental and observational evidence supporting a role for IGF‐1 signaling in CRC development 13, 14, 15, our study did not provide evidence that weight loss in individuals with obesity reduces circulating concentrations of IGF‐1. This is in line with cohort studies and other intervention studies that have demonstrated weak and inconsistent associations between intentional weight loss and changes in IGF‐1, leading some to conclude that changes in IGF‐1 may not be a major pathway through which weight changes modify cancer risk 19. We did observe an increase in the apoptotic marker, M30, following the intervention, although the change did not attain statistical significance, which may be a consequence of the limited sample size. Because a hallmark of cancer development is suppressed apoptosis, a nominal increase in apoptosis observed during the intervention supports a potential antitumorigenic effect of weight loss.

While our study suggests weight loss could mitigate the risk of CRC for individuals with obesity, some studies of bariatric surgery have demonstrated an increased risk of CRC following weight loss achieved through surgical means 7, 8, 9. Some have suggested that the increased risk of CRC following surgery may be a consequence of changes to the microbiome 8, and there is increasing interest in the role of gut microflora in obesity and cancer 30. A recent study found changes in components of the microbiome hypothesized to affect cancer risk following a change in dietary fat and fiber alongside changes in Ki‐67 in colon tissue 21. It is possible that microbiome changes may be driving the observed reductions in cell proliferation rather than changes in the insulin signaling pathway. However, the microbiome is also known to be associated with host insulin sensitivity 26, and it is therefore challenging to tease apart these related components. Future studies of the impact of weight loss on CRC risk markers may consider evaluating changes in the gut microbiome alongside other biomarkers of CRC risk.

The current study was exploratory, and therefore the sample size was small. Although we observed effects of weight loss on serologic markers of CRC risk and Ki‐67, the confidence intervals were wide. It will, therefore, be important to replicate our findings in studies with larger samples. Our small sample also meant that we were unable to explore gender differences in the observed serum and tissue changes. Links between CRC and obesity are stronger for men than for women 31; however, as with most weight loss studies 32, the majority of our sample were female. Similarly, we were not able to explore differences by baseline metabolic profile. Our study did include individuals with a “healthier obese” phenotype, i.e., individuals with BMI ≥ 30 kg/m^2^ but who did not exhibit metabolic abnormalities such as hyperinsulinemia and who may not have the same risk of CRC as those individuals with a less healthy metabolic profile 29, 30. Future studies might explore associations between improvements in metabolic health and subsequent changes in tissue markers for CRC risk.

Our sample was relatively healthy and so may not be representative of the wider population with obesity that may also have other comorbidities. Furthermore, our sample was relatively young, and CRC is typically diagnosed in older age groups. However, there have been recent increases in CRC diagnoses at younger ages, which may be a consequence of lifestyle changes at the population level 31, 32. Therefore, our observation of changes in tissue in this younger sample may be an important indicator that weight and weight changes can influence the risk of CRC from an early age.

An additional limitation of this study was the lack of a control group. Although a single‐arm design is common in biomarker‐intervention studies, a control group would help to substantiate the indication that the observed changes are a consequence of the observed weight loss. Meal replacement products not only induce weight loss, but also alter an individual's diet (e.g., the nutritional balance). Teasing out the role of dietary change versus weight change is a challenge, given these factors are somewhat inextricably linked. The previous study reporting changes in Ki‐67 following dietary change 25 did not report whether there were also any changes in weight. Future studies might seek to explore the impact of a meal replacement diet that does not reduce energy intake, and should include measures of physical activity. Furthermore, our intervention was short (8 weeks) and our participants were either still in negative energy balance or had only just reached a weight plateau. It could be important to consider whether these findings would be maintained after a period of sustained weight loss and what happens to the profile of these changes if weight is regained.

Dietary programs that typically achieve smaller amounts of weight loss are the most widely used method of weight reduction 33, 34, and the latest obesity guidance 35, 36 recommends that a loss of just 3% of body weight may have health benefits. It will therefore be important from a public health perspective to explore the changes seen at the tissue level with smaller amounts of weight loss. Future research should also seek to better understand the long‐term impact of weight loss on markers for CRC risk and to quantify the amount and duration of weight loss required for sustained benefits to an individual's CRC risk, as well as the impact of any weight regain.

Although the current study was exploratory and in a small healthy sample, it is one of the few studies to investigate this level of weight loss achieved through nonsurgical means and its effects on both serologic and colorectal tissue markers. Our findings suggest that substantial intentional weight loss achieved through a low‐energy meal replacement diet is feasible in this “healthy” population with obesity, has an impact on markers for CRC in serum, and reduces cell proliferation in colon tissue. Replicating these findings in larger samples and incorporating microbiome and other metabolic data may help us to better understand the potential impact of weight loss on risk of CRC for individuals with obesity and the mechanisms underlying the obesity‐CRC link.
